# Associations between dietary inflammatory potential and COPD: the mediating role of inflammation

**DOI:** 10.3389/fnut.2026.1762993

**Published:** 2026-05-01

**Authors:** Aimin Wang, Qingxia Cui, Weijing Meng, Qida He, Guiya Guo, Wangchen Song, Yanxia Wang, Xinyu Yang, Yonghua Ma, Na Sun, Suzhen Wang, Fuyan Shi

**Affiliations:** 1 Department of Health Statistics, School of Public Health, Shandong Second Medical University, Weifang, Shandong, China; 2 Department of Mathematical Statistics, School of Public Health, Shandong Second Medical University, Weifang, Shandong, China; 3 Department of Infectious Diseases and Public Health, City University of Hong Kong, Hong Kong SAR, China

**Keywords:** chronic obstructive pulmonary disease, dietary inflammatory index, inflammation, mediation analysis, prospective studies

## Abstract

**Background:**

Chronic obstructive pulmonary disease (COPD) continues to pose a significant global burden, which may be exacerbated by pro-inflammatory diets. This study aimed to investigate the association between the Dietary Inflammatory Index (DII) and the Energy-adjusted Dietary Inflammatory Index (E-DII) and the risk of COPD.

**Methods:**

A total of 167,440 participants were recruited for a prospective analysis from the UK Biobank. Cox proportional hazards models were employed to analyze the associations of DII and E-DII with COPD. Restricted cubic spline (RCS) and subgroup analyses were also performed. Additionally, mediation analyses were conducted to explore the potential mediating role of inflammatory biomarkers between DII, E-DII and COPD risk.

**Results:**

During a median follow-up of 13.4 years, 4,041 COPD cases occurred. After adjusting for covariates, each unit increase in DII and E-DII was associated with a 5% [HR: 1.05 (95% CI: 1.04–1.07)] and 20% [HR: 1.20 (95% CI: 1.16–1.25)] higher risk of COPD. Participants in the highest quartile of DII and E-DII scores had a higher risk of COPD compared to those in the lowest quartile, with HR = 1.27 (95% CI: 1.17–1.39) and HR = 1.42 (95% CI: 1.30–1.55). In mediation analyses, C-reactive protein (CRP) showed the highest proportion mediated for both DII and E-DII, followed by systemic immune-inflammation index (SII). Subgroup analyses indicated population heterogeneity, and sensitivity analyses confirmed the robustness of the findings.

**Conclusion:**

Both DII and E-DII are associated with an increased risk of COPD, and this association may be partially explained by systemic inflammation, particularly CRP and SII.

## Introduction

Chronic obstructive pulmonary disease (COPD) is a chronic respiratory condition caused by abnormalities in the airways and alveolar damage, typically characterized by persistent and progressive symptoms (1). According to the 2019 Global Burden of Disease (GBD) report, more than 350 million people worldwide suffer from COPD, with approximately 3.2 million deaths each year, making it the third leading cause of death globally and a major contributor to the global burden of disease (2). With the ongoing trends of population aging, urbanization, and environmental pollution, the incidence and mortality rates of COPD are expected to continue rising (3). Therefore, it is important to identify the risk factors for COPD and implement effective prevention strategies.

Dietary inflammatory index (DII) is a tool designed to quantify the inflammatory potential of an individual's diet, and provides an effective way to explore the association between the inflammatory effects of diet and various diseases (4). The energy-adjusted dietary inflammatory index (E-DII) assesses the inflammatory potential of an individual's diet per 1,000 kcal of energy intake, effectively controlling for confounding due to total energy consumption (5). Previous studies have shown that higher DII and E-DII scores are associated with an increased risk of various chronic diseases, including cardiovascular diseases, diabetes, neurological disorders, and certain cancers (6–10). Although several studies have reported associations between higher DII scores and adverse respiratory outcomes, most of this evidence has been derived from cross-sectional analyses or has not considered the timing of disease onset (11–13). At present, prospective evidence on the associations between DII, E-DII, and COPD is lacking.

In recent years, accumulating evidence has highlighted the critical role of inflammation in the pathogenesis of COPD (14–16). Patients with COPD often exhibit systemic inflammatory responses, as indicated by elevated levels of biomarkers such as C-reactive protein (CRP) and the systemic immune-inflammation index (SII), suggesting that inflammation in COPD is not confined to the lungs but may also be influenced by exogenous factors (17–20). As a modifiable lifestyle factor, diet has been shown to play an important role in regulating systemic inflammation (21–25). However, the potential mediating role of inflammation in the relationship between DII, E-DII and COPD risk remains unclear.

The objectives of this prospective cohort study were to (1) examine the associations between DII, EDII scores and the risk of COPD based on the UK Biobank cohort and (2) determine whether systemic inflammatory markers mediated these associations.

## Methods

### Study population

The data used in this study were obtained from the UK Biobank (Project ID 78500). The database that recruited over 500,000 participants aged 37–73 years between 2006–2010, was a large-scale prospective cohort study (26). At one of the 22 recruiting centers in the UK, participants underwent baseline physical measurements, biochemical blood tests, genetic testing, and other assessments. Information on the participants' sociodemographic characteristics, lifestyle, early life exposures, and psychosocial factors was also collected through questionnaires and personal interviews (27, 28). Details on data collection and descriptions can be found on the UK Biobank website (www.ukbiobank.ac.uk).

From the 502,411 participants initially recruited by the UK Biobank, we excluded 291,432 individuals who did not have 24-h dietary assessments. We also excluded 2,460 participants diagnosed with COPD at baseline and 41,034 participants with missing information on covariates. Finally, 167,440 participants were eligible to participate in this study (Supplementary Figure S1). For the mediation analysis, participants with missing inflammatory biomarker data or extreme outlier values (defined as values beyond 3 standard deviations from the mean) were excluded, leaving a final sample of 145,442 participants.

### Dietary inflammatory index

We assessed dietary intake using data from the UK Biobank, collected through the Oxford WebQ, a web-based 24-h dietary questionnaire. This tool captures consumption information for 206 food items and 32 beverages over the previous 24 h (29, 30). Its reliability has been confirmed in multiple validation studies.

Participants were invited to complete the questionnaire at baseline and on four separate occasions, beginning with the initial administration at the assessment center (between April 2009 and September 2010) and concluding with the final round (from April 2012 to June 2012) (31). In this study, the average nutrient intake across all available time points was used to calculate each participant's DII. Nutrient intake data obtained from 24-h dietary recalls were standardized using a global reference database, which provided the mean and standard deviation for each dietary component. A z-score was calculated for each component by subtracting the global mean from the individual intake and dividing by the global standard deviation. To minimize the effect of right skewness, the z-scores were converted to centered percentile scores and then were multiplied by literature-derived inflammatory effect scores for each dietary component, reflecting their pro- or anti-inflammatory properties. The resulting scores were summed to generate the total DII score, with a higher score indicating a pro-inflammatory diet and a lower score indicating an anti-inflammatory diet (4). For the calculation of the E-DII, we referred to previous studies (5, 32, 33). The foods and its nutrients were first adjusted for total energy intake (density method = nutrient (food)/total energy intake × 1,000 kcal). Then, the steps similar to the DII calculation were repeated to obtain the E-DII. To facilitate further analysis, the DII and E-DII scores were evenly divided into quartiles, ensuring a balanced classification of dietary inflammatory potential across the study population. Due to data availability, the DII and E-DII were calculated based on 29 dietary parameters available in the UK Biobank dataset (Supplementary Table S1).

### Ascertainment of outcome

The outcome in this study was COPD, identified using the International Classification of Diseases 10th Revision (ICD-10) codes J40–J44. Diagnosis information was obtained predominantly from primary care records, hospital admissions, and self-reported health conditions. The earliest recorded diagnosis date of COPD was defined as the event date in this study. The follow-up period for this study ended on 1 January 2023. The follow-up time was defined as the time from the recruitment date to death or the end of the current follow-up, with a median duration of 13.4 years.

### Assessment of covariates

At baseline, we recorded participants' sociodemographic information, behavioral factors and health-related information as covariates. Sociodemographic information included age (years), sex (female or male), ethnicity (white and other ethnic groups), education (college/university degree or other) and the Townsend deprivation index (TDI). Behavioral factors included smoking status (never, previous, or current smoking), alcohol intake frequency (never or special occasions only, once to three times per month, once to twice times per week, three to four times per week, and daily or almost daily), physical activity (yes or no, indicating whether a person met the 2017 UK physical activity guidelines, defined as ≥150 min of moderate-intensity activity per week or ≥75 min of vigorous-intensity activity) and sleep duration (hours). Health-related information included body mass index (BMI, kg/m^2^), hypertension (yes or no), diabetes (yes or no), hyperlipidemia (yes or no) and asthma (yes or no).

### Inflammatory biomarkers

To investigate the role of inflammation in the associations between DII, E-DII and COPD, we selected several well-established inflammatory biomarkers, including C-reactive protein (CRP), neutrophil-to-lymphocyte ratio (NLR), platelet-to-lymphocyte ratio (PLR), systemic immune-inflammation index (SII), and lymphocyte-to-monocyte ratio (LMR). Peripheral blood samples were collected at baseline and analyzed at the UK Biobank central laboratory within 24 h of collection. Hematological indicators, including neutrophil, lymphocyte, monocyte, and platelet counts were measured using a Beckman Coulter LH750 Hematology Analyzer. Based on these measurements, inflammatory indices were calculated as follows: NLR (neutrophils/lymphocytes), PLR (platelets/lymphocytes), LMR (lymphocytes/monocytes), and SII (neutrophils × platelets/lymphocytes). Serum CRP concentrations were determined using a high-sensitivity immunoturbidimetric assay on a Beckman Coulter AU5800 analyzer.

### Statistical analysis

Continuous variables were presented as medians (25th and 75th percentiles), and differences between groups were compared using the Mann-Whitney *U*-test. Categorical variables were presented as frequencies and percentages, and differences between different groups were compared using the chi-square test. Cox proportional hazards models were used to examine the association between DII, E-DII and the risk of COPD. Results are expressed by hazard ratios (HRs) and 95% confidence intervals (CIs). To adjust for various potential confounding factors, we sequentially constructed four different Cox regression models: Model 1 involved only univariate analysis based on DII or E-DII. Model 2 additionally adjusted for sociodemographic factors, including age, sex, ethnicity, education, and TDI on the basis of Model 1. Model 3 further incorporated behavioral factors into Model 2, including smoking status, alcohol intake frequency, physical activity and sleep duration. Model 4 additionally adjusted for Health-related information (BMI, hypertension, diabetes, hyperlipidemia and asthma) on the basis of Model 3. The proportional risk hypothesis for each Cox regression model was verified using Schoenfeld residuals and all tested hypotheses passed. Additionally, a linear trend test was conducted by including each DII and E-DII quartile as a continuous variable in the regression model. To assess potential non-linear relationships, restricted cubic spline (RCS) models with 3 knots (5th, 50th, and 95th) were used to explore the dose-response associations between DII, E-DII and the risk of COPD.

We also conducted subgroup analyses stratified by age (< 60 or ≥60 years), sex, ethnicity, education, and BMI (< 25 or ≥25 kg/m^2^), and tested for interactions between these subgroup variables and DII or E-DII using likelihood ratio tests.

In our study, survival mediation analyses were performed using the ‘CMAverse' package in R to assess whether inflammatory biomarkers mediated the associations of DII and E-DII with incident COPD. This approach is based on the counterfactual framework and allows for decomposition of the total effect into natural direct effects (NDE) and natural indirect effects (NIE) (34). The mediation models were adjusted for potential confounders, including age, sex, ethnicity, education, TDI, smoking status, alcohol intake frequency, physical activity and sleep duration.

Furthermore, we also did some additional analysis to test the robustness of the findings: (1) To reduce the potential reverse causality, we excluded participants diagnosed with COPD within the first 2 years of follow-up. (2) The Fine-Gray competing risk model was used to accommodate competing risks from deaths before the incidence of COPD. (3) Participants with asthma at baseline were excluded to mitigate the potential bias resulting from these medical histories. (4) To address missing covariate data, multiple imputation was performed under the missing at random assumption.

All statistical analyses were carried out using R (version 4.4.3). Statistical significance was set at a 2-tailed threshold of *P* < 0.05 throughout the study.

## Results

### Characteristics of study population

The baseline characteristics of the study population are displayed in Table 1. The final analytic sample comprised 167,440 participants with a median age of 57 (49, 62) years. During a median follow-up period of 13.4 years, 4,041 individuals were diagnosed with COPD. The DII and E-DII values were higher in COPD patients compared with participants without COPD. COPD patients were more likely to be older, male, and to have higher TDI and BMI. They also exhibited a higher prevalence of hypertension, diabetes, hyperlipidemia, and asthma (*P* < 0.05). Additionally, significant differences between the COPD and non-COPD groups were observed in ethnicity, educational level, smoking status, alcohol intake frequency, and physical activity (*P* < 0.05).

**Table 1:** Characteristics of the study participants categorized by the incidence of COPD.

Characteristics	Overall (*N* = 167,440)	Non-COPD (*N* = 163,399)	COPD (*N* = 4,041)	*P-*value
DII	−0.46 (−1.85, 0.91)	−0.47 (−1.85, 0.90)	−0.17 (−1.71, 1.30)	< 0.001
E-DII	0.19 (−0.35, 0.71)	0.18 (−0.35, 0.70)	0.34 (−0.25, 0.92)	< 0.001
Age, years	57 (49, 62)	57 (49,62)	62 (58,66)	< 0.001
Sex, *n* (%)				
Female	89,042 (53.2)	87,328 (53.4)	1,714 (42.4)	< 0.001
Male	78,398 (46.8)	76,071 (46.6)	2,327 (57.6)	
Ethnicity, *n* (%)				
White	160,612 (95.9)	156,664 (95.9)	3,948 (97.7)	< 0.001
Others	6,828 (4.1)	6,735 (4.1)	93 (2.3)	
Education, *n* (%)				
College or University degree	75,790 (45.3)	74,684 (45.7)	1,106 (27.4)	< 0.001
Others	91,650 (54.7)	88,715 (54.3)	2,935 (72.6)	
TDI	−2.35 (−3.75, 0.00)	−2.37 (−3.75, −0.03)	−1.70 (−3.50, 1.19)	< 0.001
Smoking status, *n* (%)				
Never	95,216 (56.9)	94,344 (57.7)	872 (21.6)	< 0.001
Former	59,479 (35.5)	57,428 (35.1)	2,051 (50.7)	
Current	12,745 (7.6)	11,627 (7.2)	1,118 (27.7)	
Alcohol intake frequency, *n* (%)				
Never or special occasions only	25,235 (15.1)	24,339 (14.9)	896 (22.2)	< 0.001
One to three times per month	18,040 (10.8)	17,648 (10.8)	392 (9.7)	
Once to twice per week	41,532 (24.8)	40,703 (24.9)	829 (20.5)	
Three or four times per week	43,074 (25.7)	42,319 (25.9)	755 (18.7)	
Daily or almost daily	39,559 (23.6)	38,390 (23.5)	1,169 (28.9)	
Physical activity, *n* (%)				
No	29,881 (17.8)	29,004 (17.8)	877 (21.7)	< 0.001
Yes	137,559 (82.2)	134,395 (82.2)	3,164 (78.3)	
Sleep duration, hours	7 (7, 8)	7.0 (7, 8)	7.0 (6, 8)	0.590
BMI, kg/m^2^	26.17 (23.71, 29.17)	26.14 (23.70, 29.12)	27.32 (24.39, 30.69)	< 0.001
Hypertension, *n* (%)				
No	127,880 (76.4)	125,478 (76.8)	2,402 (59.4)	< 0.001
Yes	39,560 (23.6)	37,921 (23.2)	1,639 (40.6)	
Diabetes, *n* (%)				
No	160,827 (96.1)	157,160 (96.2)	3,667 (90.7)	< 0.001
Yes	6,613 (3.9)	6,239 (3.8)	374 (9.3)	
Hyperlipidemia, *n* (%)				
No	145,362 (86.8)	142,335 (87.1)	3,027 (74.9)	< 0.001
Yes	22,078 (13.2)	21,064 (12.9)	1,014 (25.1)	
Asthma, *n* (%)				
No	149,308 (89.2)	146,365 (89.6)	2,943 (72.8)	< 0.001
Yes	18,132 (10.8)	17,034 (10.4)	1,098 (27.2)	

BMI, body mass index; COPD, chronic obstructive pulmonary disease; DII, dietary inflammatory index; E-DII, energy-adjusted dietary inflammatory index; TDI, Townsend deprivation index.

### Association between DII, EDII and the risk of COPD

Table 2 presents the relations of DII and EDII scores with the risk of COPD. After adjusting for multiple covariates (Model 4), each one-unit increase in DII was associated with a hazard ratio (HR) of 1.05 (95% CI: 1.04–1.07, *P* < 0.001) for COPD, while each one-unit increase in E-DII was associated with an HR of 1.20 (95% CI: 1.16–1.25, *P* < 0.001). In addition, when DII and E-DII were analyzed as categorical variables, higher levels (Q4 vs. Q1) were significantly associated with an increased risk of COPD. Specifically, participants in the highest DII quartile had a hazard ratio (HR) of 1.27 (95% CI: 1.17–1.39, *P* < 0.001), while those in the highest E-DII quartile had an HR of 1.42 (95% CI: 1.30–1.55, *P* < 0.001), after adjusting for a range of influencing factors. Furthermore, trend analysis indicated a significant positive association between DII, E-DII and the risk of COPD (*P* for trend < 0.001).

**Table 2:** Association between DII, E-DII and the risk of COPD.

Index	Model 1		Model 2		Model 3		Model 4	
	HR (95% CI)	*P*-value	HR (95% CI)	*P*-value	HR (95% CI)	*P*-value	HR (95% CI)	*P*-value
DII								
Continuous	1.08 (1.06, 1.09)	< 0.001	1.10 (1.08, 1.12)	< 0.001	1.06 (1.04, 1.07)	< 0.001	1.05 (1.04, 1.07)	< 0.001
Quartile								
Q1	1 (Reference)		1 (Reference)		1 (Reference)		1 (Reference)	
Q2	0.94 (0.85, 1.03)	0.155	1.01 (0.92, 1.11)	0.827	1.00 (0.91, 1.10)	0.994	1.01 (0.92, 1.11)	0.807
Q3	1.03 (0.94, 1.13)	0.482	1.13 (1.03, 1.24)	0.008	1.04 (0.95, 1.14)	0.421	1.04 (0.95, 1.14)	0.420
Q4	1.37 (1.26, 1.49)	< 0.001	1.55 (1.43, 1.69)	< 0.001	1.29 (1.18, 1.41)	< 0.001	1.27 (1.17, 1.39)	< 0.001
*P* for trend		< 0.001		< 0.001		< 0.001		< 0.001
E-DII								
Continuous	1.30 (1.25, 1.35)	< 0.001	1.37 (1.32, 1.42)	< 0.001	1.22 (1.17, 1.27)	< 0.001	1.20 (1.16, 1.25)	< 0.001
Quartile								
Q1	1 (Reference)		1 (Reference)		1 (Reference)		1 (Reference)	
Q2	1.01 (0.92, 1.11)	0.874	1.05 (0.95, 1.15)	0.323	1.03 (0.94, 1.13)	0.548	1.02 (0.93, 1.12)	0.691
Q3	1.08 (0.99, 1.19)	0.09	1.16 (1.06, 1.28)	0.001	1.08 (0.98, 1.19)	0.103	1.07 (0.98, 1.18)	0.132
Q4	1.62 (1.49, 1.76)	< 0.001	1.79 (1.64, 1.95)	< 0.001	1.45 (1.33, 1.59)	< 0.001	1.42 (1.30, 1.55)	< 0.001
*P* for trend		< 0.001		< 0.001		< 0.001		< 0.001

Hazard ratio for per increase of 1 in DII or E-DII. Model 1 represents the single-factor Cox regression model (DII or E-DII). Model 2 adjusted model 1 + age, sex, ethnicity, education, and TDI. Model 3 adjusted model 2 + smoking status, alcohol intake frequency, physical activity and sleep duration. Model 4 adjusted model 3 + BMI, hypertension, diabetes, hyperlipidemia and asthma. CI, confidence interval; DII, dietary inflammatory index; E-DII, energy-adjusted dietary inflammatory index; HR, hazard ratio; Q, quartile.

**Figure 1:**
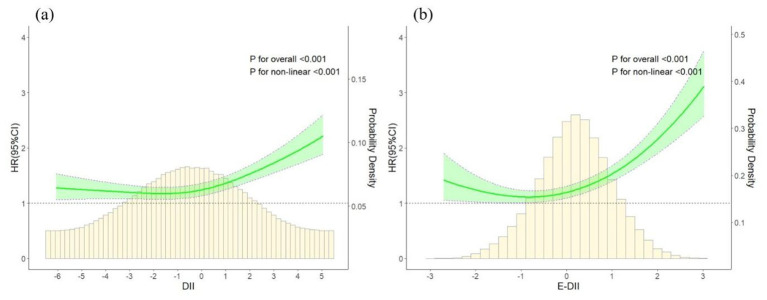
Non-linear associations between **(a)** DII, **(b)** E-DII, and risk of COPD. Hazard ratios were based on Cox regression models adjusted for age, sex, ethnicity, education, TDI, smoking status, alcohol intake frequency, physical activity, sleep duration, BMI, hypertension, diabetes, hyperlipidemia and asthma. CI, confidence interval; HR, hazard ratio.

Restricted cubic spline models demonstrated J-shaped non-linear relationships of DII and E-DII with COPD risk (*P* for non-linear < 0.001) (Figure 1). Additionally, we found that when DII and E-DII were below 0.5, the risk of COPD remained relatively stable, but when they exceeded 0.5, the risk of COPD increased significantly.

### Subgroup analyses

**Figure 2:**
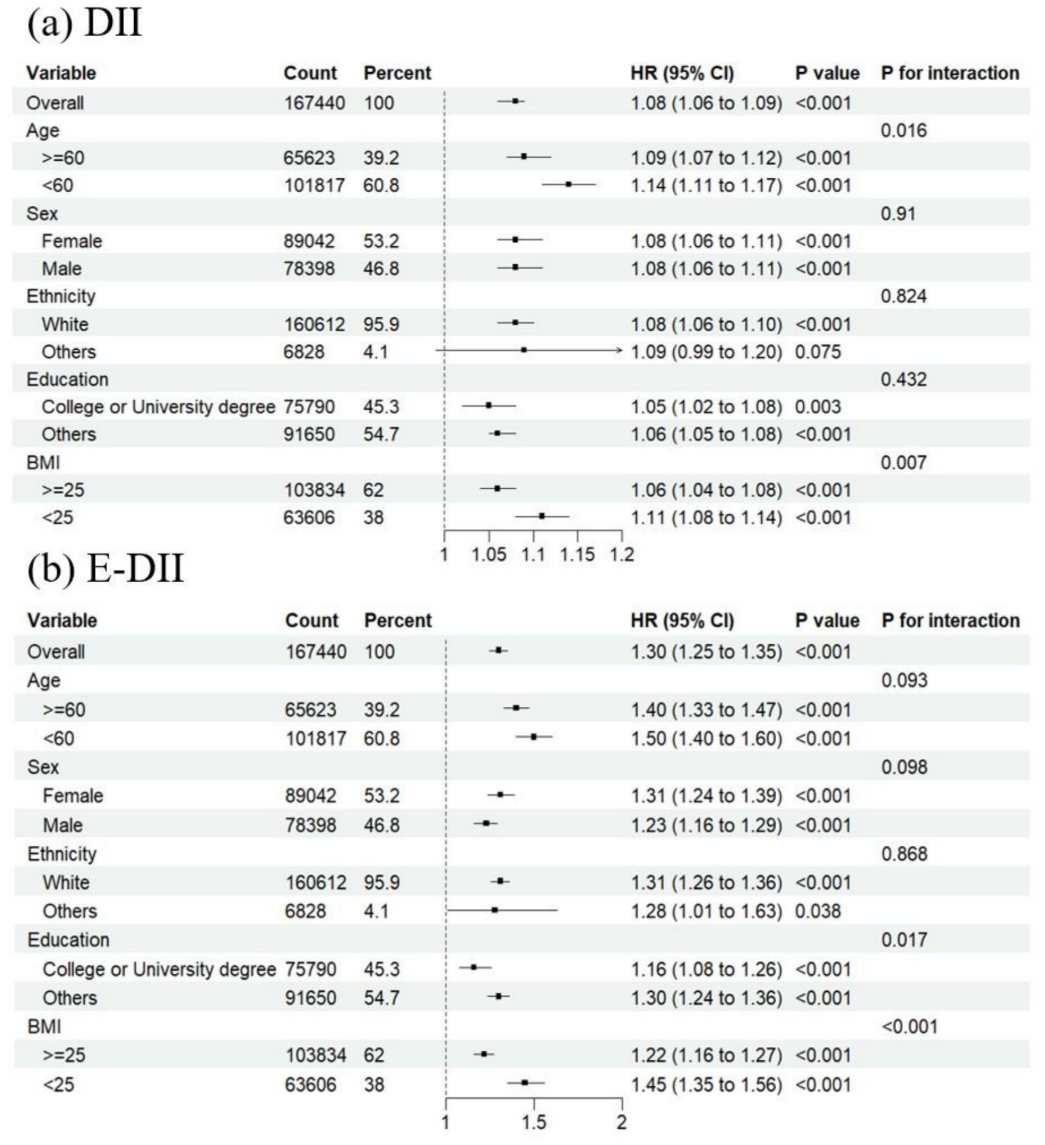
Associations between **(a)** DII, **(b)** E-DII, and COPD across different subgroups. DII, dietary inflammatory index; E-DII, energy-adjusted dietary inflammatory index; BMI, body mass index.

In the subgroup analyses (Figure 2), a significant interaction between DII, E-DII and BMI was observed (*P* for interaction < 0.05), with stronger associations among participants with a BMI < 25 kg/m^2^. Moreover, the effect of E-DII on COPD risk appeared to be more pronounced in individuals with lower educational attainment compared to those with a college or university degree. No significant effect modifications were observed in the remaining subgroup analyses (*P* for interaction > 0.05).

### Mediation analyses

In the mediation analysis, CRP showed the highest proportion mediated (PM) for both DII (PM = 9.40%, 95% CI: 6.59%−14.63%) and E-DII (PM = 7.92%, 95% CI: 6.17%−10.52%). This was followed by SII, with a PM of 3.23% (95% CI: 1.96%−5.75%) for DII and 3.11% (95% CI: 2.06%−4.38%) for E-DII. For NLR, a relatively small mediating effect was observed only for E-DII (PM = 1.26%, 95% CI: 0.76%−1.93%), whereas no significant mediation was detected for DII. In contrast, PLR showed no evidence of mediation in either model. Notably, LMR demonstrated a negative mediation proportion for DII (PM = −1.27%, 95% CI: −2.38%–−0.52%) (Table 3).

**Table 3:** Mediation analysis of inflammation in the association between DII/E-DII and COPD risk.

Inflammatory biomarkers	Natural direct effect	Natural indirect effect	Proportion mediated, % (95% CI)
	HR (95% CI)	HR (95% CI)	
DII			
SII	1.050 (1.030, 1.069)^*^	1.002 (1.001, 1.002)^*^	3.23 (1.96, 5.75)^*^
NLR	1.051 (1.032, 1.073)^*^	1.000 (0.999, 1.001)	0.51 (−0.11, 1.33)
PLR	1.051 (1.031, 1.072)^*^	1.000 (0.999, 1.000)	−0.14 (−0.61, 0.26)
LMR	1.052 (1.035, 1.072)^*^	0.999 (0.999, 0.999)^*^	−1.27 (−2.38, −0.52)^*^
CRP	1.046 (1.028, 1.067)^*^	1.005 (1.003, 1.005)	9.40 (6.59, 14.63)^*^
E–DII			
SII	1.204 (1.153, 1.259)^*^	1.005 (1.003, 1.007)^*^	3.11 (2.06, 4.38)^*^
NLR	1.208 (1.157, 1.263)^*^	1.002 (1.001, 1.003)^*^	1.26 (0.76, 1.93)^*^
PLR	1.211 (1.158, 1.267)^*^	1.000 (0.999, 1.000)	−0.19 (−0.53, 0.05)
LMR	1.212 (1.159, 1.263)^*^	1.000 (0.999, 1.000)	−0.16 (−0.48, 0.13)
CRP	1.192 (1.139, 1.248)^*^	1.014 (1.012, 1.016)^*^	7.92 (6.17, 10.52)^*^

Mediation analysis was adjusted for various factors, including age, sex, ethnicity, education, TDI, smoking status, alcohol intake frequency, physical activity, sleep duration. DII, dietary inflammatory index; E-DII, energy-adjusted dietary inflammatory index; SII, systemic immune-inflammation index; NLR, neutrophil-to-lymphocyte ratio; PLR, platelet-to-lymphocyte ratio; LMR, lymphocyte-to-monocyte ratio; CRP, C-reactive protein. ^*^: *P* < 0.05.

### Sensitivity analyses

After excluding participants diagnosed with COPD within the first 2 years of follow-up, similar results were observed in the overall population (Supplementary Table S2). In the competing risk models, where death was considered as a competing event, the associations remained statistically significant (Supplementary Table S3). In addition, after excluding participants who already had asthma at baseline, the results still showed no significant changes (Supplementary Table S4). After multiple imputation of missing covariate data, the results remained largely unchanged (Supplementary Table S5).

## Discussion

This study investigated the association between DII, E-DII and the risk of COPD in a large prospective cohort of 167,440 participants. The main findings demonstrated a significant positive association between higher DII and E-DII scores and increased COPD risk. Compared with individuals in the lowest quartile, those in the highest quartile of DII had a 27% higher risk of COPD, while those in the highest quartile of E-DII had a 42% higher risk. These associations were further confirmed in sensitivity analyses. In addition, we identified a mediating role of inflammatory biomarkers in the association between DII, E-DII and COPD risk. Overall, our findings suggest that a more pro-inflammatory diet is associated with a higher risk of COPD, highlighting the potential role of dietary inflammation in COPD prevention.

Most cases of COPD can be prevented by managing modifiable risk factors, with diet being one of the most accessible and cost-effective lifestyle interventions (35–40). An increasing number of studies have shown that different dietary patterns are closely associated with the risk of developing COPD (38–40). For instance, consuming a large number of fruits and vegetables over a long period of time can effectively reduce the risk of developing COPD, and this effect is particularly pronounced among smokers (41, 42). Individuals with lower alcohol intake (1–30 g/day) had a lower risk of developing COPD compared to no consumers (43). By contrast, heavy alcohol intake has been proven to have a negative impact on lung function (44). Ultra-processed food consumption was also linked to an increased risk of chronic respiratory diseases (45). Additionally, a higher dietary approaches to stop hypertension (DASH) diet score was associated with improved COPD prevalence, lung function and respiratory symptoms (46). In the above-mentioned studies (38–40), it has been observed that the intake of anti-inflammatory foods may help reduce the risk of developing COPD. Most foods and nutrients can trigger either pro-inflammatory or anti-inflammatory responses in the body, which in turn influence the risk of COPD onset (38). DII and E-DII, as tools for assessing the overall inflammatory potential of an individual's diet, have been shown to be associated with an increased risk of various chronic diseases when their scores are elevated (4, 5). Two recent cross-sectional studies based on the NHANES database also found that high DII values were associated with an increased risk of COPD (12, 13). Our longitudinal study results not only confirmed this observation but also expanded it by demonstrating that the diet inflammatory index after energy-adjusted dietary inflammatory index was more strongly associated with the risk of COPD. This finding strengthens the potential value of DII and E-DII as modifiable dietary indicators in COPD prevention. The stronger association observed for E-DII may be attributable to its adjustment for total energy intake, as DII may be influenced by overall energy consumption, potentially introducing confounding (5). In contrast, E-DII reflects the inflammatory density of the diet and may therefore provide a more precise estimate of dietary inflammatory potential.

Several mechanisms may underlie the associations between DII, E-DII and the risk of COPD. First, systemic inflammation is likely to play a central role. Diets with higher inflammatory potential may promote chronic low-grade inflammation, which is closely related to airway remodeling, impaired lung function, and the pathogenesis of COPD. In this process, inflammatory biomarkers such as CRP and SII may play key roles, supporting the hypothesis that systemic inflammation partially mediates the relationship between dietary patterns and COPD onset. In our study, a negative mediation effect was observed for LMR in the association between DII and COPD, whereas no significant mediation was identified for E-DII. This pattern may indicate a suppression effect or reflect complex interactions among immune cell components, and could also arise from model instability or residual confounding. Second, pro-inflammatory diet may increase oxidative stress, thereby exacerbate alveolar destruction and reduce lung function (47). Pro-inflammatory dietary patterns are often associated with metabolic disturbances, including obesity (48), hyperlipidemia (49), and metabolic syndrome (50), which are all linked to increased COPD risk (51–53). Finally, some anti-inflammatory nutrients (such as ω-3 fatty acids, ω-6 fatty acids, dietary fibers, vitamin C and E, etc.) are believed to have antioxidant and anti-inflammatory effects (54–57), which can help slow down the progression of COPD. Overall, these mechanisms support the role of anti-inflammatory dietary patterns in the prevention and management of COPD.

Additionally, lower DII and E-DII levels were also associated with the occurrence of COPD, indicating a J-shaped relationship in which both high and very low levels may be linked to increased risk. Very low DII or E-DII values may reflect restrictive or nutritionally imbalanced diets, such as insufficient energy intake or limited dietary diversity, which could impair immune function and increase vulnerability to respiratory diseases. Furthermore, individuals with underlying health conditions or heightened susceptibility may be more likely to adopt healthier or anti-inflammatory dietary patterns. Therefore, these findings warrant further validation in future studies.

### Strengths and limitations

Several strengths of this study are important to highlight. First, the use of a large prospective cohort with extended follow-up allowed us to evaluate incident COPD and establish temporal ordering between exposure and outcome. Second, we further examined the potential non-linear (J-shaped) relationships between DII, E-DII and risk of COPD. Third, we also examined the impact of DII and E-DII on COPD in different subgroups of the population. Fourth, we conducted a thorough analysis and found of some inflammatory factors (such as CRP, SII, and NLR) may serve as important mediators in the association between DII, E-DII and COPD onset. Finally, several sensitivity analyses were carried out, which verified the robustness of the results.

Nonetheless, it is important to acknowledge several inherent limitations to our study. First, this is an observational study, so it cannot establish a causal relationship between DII, E-DII and the risk of COPD. Second, dietary intake was self-reported and assessed at limited time points, which may introduce measurement error and may not reflect long-term dietary patterns, potentially leading to time-varying exposure bias. Third, the calculation of DII and E-DII scores was limited by the lack of complete information on several dietary components. Fourth, although we adjusted for a wide range of potential confounding factors, the possibility of residual confounding cannot be ruled out. Finally, the majority of participants in the UK Biobank were from the UK and predominantly of White ethnicity, which may limit the generalizability of our findings to other populations or ethnic groups.

## Conclusion

In conclusion, this study found that high DII and E-DII were associated with an increased risk of COPD, and this association may be partially explained by systemic inflammation. These results highlight the potential of anti-inflammatory dietary patterns as a preventive strategy against COPD, with implications for both disease prevention and adjunctive treatment in clinical management.

## Data Availability

Publicly available datasets were analyzed in this study. This data can be found here: https://www.ukbiobank.ac.uk UK Biobank Application Number 78500.
