# Supplementary material for: Associations between dietary inflammatory potential and COPD: the mediating role of inflammation
Source: Front Nutr. 2026 May 1;13:1762993. doi: 10.3389/fnut.2026.1762993 (PMC13175968; doi:10.3389/fnut.2026.1762993)
Supplement: Supplementary file 1 [file Table_1.docx]

**Associations between dietary inflammatory potential and COPD: mediating roles of inflammation**

Aimin Wang^1*^, Qingxia Cui^2*^, Weijing Meng^1*^, Qida He^3^, Guiya Guo^1^, Wangchen Song^1^, Yanxia Wang^1^, Xinyu Yang^1^, Yonghua Ma^1^, Na Sun^1^, Suzhen Wang^1^, and Fuyan Shi^1^

^1^Department of Health Statistics, School of Public Health, Shandong Second Medical University, Weifang, Shandong 261053, China

^2^Department of Mathematical Statistics, School of Public Health, Shandong Second Medical University, Weifang, Shandong 261053, China

^3^Department of Infectious Diseases and Public Health, City University of Hong Kong, Hong Kong SAR, China.

^*^These authors contributed equally to this study and are co-first authors.

**Correspondence:**

Fuyan Shi, School of Public Health, Shandong Second Medical University, Weifang, Shandong 261053, China.

Email: shifuyan@sdsmu.edu.cn

**Contents**

[**Supplementary Fig 1** Flowchart of study population inclusion and study design 3](#_Toc226880612)

[**Supplementary Table 1.** Foods items for calculating the dietary inflammatory index. 4](#_Toc226880613)

[**Supplementary Table 2.** The association of DII, E-DII with COPD risk after excluding participants who experienced a COPD within the first two years of follow-up (N = 167,238). 5](#_Toc226880614)

[**Supplementary Table 3.** Association between DII, E-DII and risk of COPD using competitive risk model (N = 167,440). 6](#_Toc226880615)

[**Supplementary Table 4.** The association of DII, E-DII with COPD risk after excluding participants with asthma at baseline (N = 149,308). 7](#_Toc226880616)

[**Supplementary Table 5.** Association of DII and E-DII with the risk of COPD after multiple imputation of missing covariates (N = 208,255). 8](#_Toc226880617)


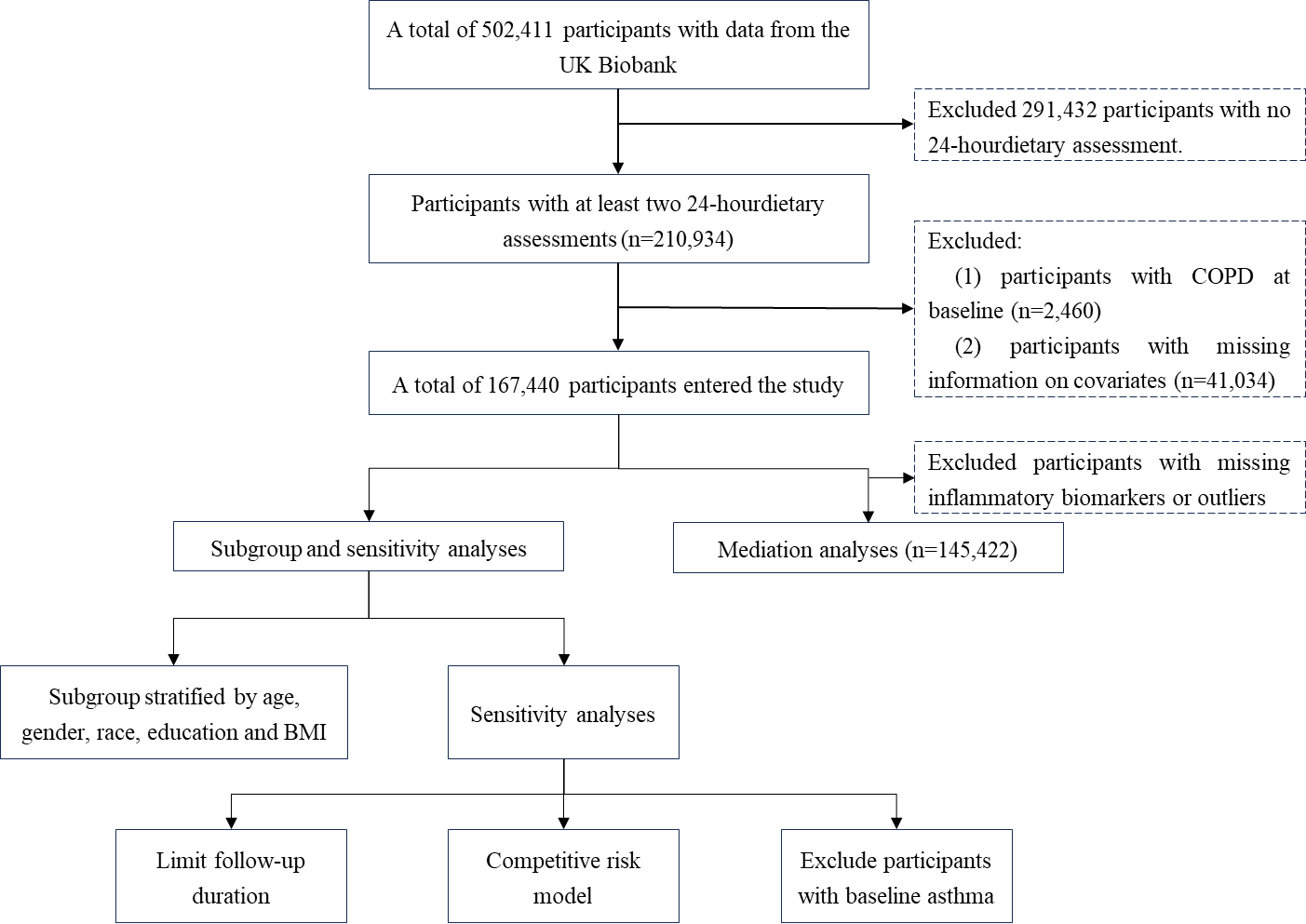


**Supplementary Fig 1** Flowchart of study population inclusion and study design

**Supplementary Table 1.** Foods items for calculating the dietary inflammatory index.

| Group | Food parameter | Code | Inflammatory effect score | Global daily mean intake(units/day) |
| --- | --- | --- | --- | --- |
| Pro-inflammation | Saturated fat (g/day) | 26014 | 0.373 | 28.6 ± 8.0 |
|  | Total fat (g/day) | 26008 | 0.298 | 71.4 ± 19.4 |
|  | Trans fatty acid (g/day) | 26155 | 0.229 | 3.15 ± 3.75 |
|  | Energy (kcal/day) | 26002 | 0.180 | 2056 ± 338 |
|  | Cholesterol (mg/day) | 26037 | 0.110 | 279.4 ± 51.2 |
|  | Vitamin B12 (μg/day) | 26021 | 0.106 | 5.15 ± 2.70 |
|  | Carbohydrate (g/day) | 26013 | 0.097 | 272.2 ± 40.0 |
|  | Iron (mg/day) | 26019 | 0.032 | 13.35 ± 3.71 |
|  | Total Protein (g/day) | 26005 | 0.021 | 79.4 ± 13.9 |
| Anti-inflammation | Dietary fiber (g/day) | 26017 | -0.663 | 18.8 ± 4.9 |
|  | β-Carotene (μg/day) | 26039 | -0.584 | 3718 ± 1720 |
|  | Tea (g/day) | 26141 | -0.536 | 1.69 ± 1.53 |
|  | Mg (mg/day) | 26025 | -0.484 | 310.1 ± 139.4 |
|  | Vitamin D (μg/day) | 26029 | -0.446 | 6.26 ± 2.21 |
|  | ω-3 fatty acids (g/day) | 26015 | -0.436 | 1.06 ± 1.06 |
|  | Vitamin C (mg/day) | 26023 | -0.424 | 118.2 ± 43.46 |
|  | Vitamin E (mg/day) | 26028 | -0.419 | 8.73 ± 1.49 |
|  | Vitamin A (μg/day) | 26061 | -0.401 | 983.9 ± 518.6 |
|  | Vitamin B6 (mg/day) | 26020 | -0.365 | 1.47 ± 0.74 |
|  | Polyunsaturated fat (g/day) | 100007 | -0.337 | 13.88 ± 3.76 |
|  | Zinc (mg/day) | 26033 | -0.313 | 9.84 ± 2.19 |
|  | Alcohol (g/day) | 26030 | -0.278 | 13.98 ± 3.72 |
|  | Niacin (mg/day) | 26054 | -0.246 | 25.90 ± 11.77 |
|  | Selenium (μg/day) | 26058 | -0.191 | 67.0 ± 25.1 |
|  | Folate (μg/day) | 26022 | -0.190 | 273.0 ± 70.7 |
|  | ω-6 fatty acids (g/day) | 26016 | -0.159 | 10.80 ± 7.50 |
|  | Thiamin (mg/day) | 26034 | -0.098 | 1.70 ± 0.66 |
|  | Riboflavin (mg/day) | 26035 | -0.068 | 1.70 ± 0.79 |
|  | Monounsaturated fat (g/day) | 26032 | -0.009 | 27.0 ± 6.1 |

**Supplementary Table 2.** The association of DII, E-DII with COPD risk after excluding participants who experienced a COPD within the first two years of follow-up (N = 167,238).

| Index | Model 1 | |  | Model 2 | |  | Model 3 | |  | Model 4 | |
| --- | --- | --- | --- | --- | --- | --- | --- | --- | --- | --- | --- |
|  | HR (95% CI) | *P*-value |  | HR (95% CI) | *P*-value |  | HR (95% CI) | *P*-value |  | HR (95% CI) | *P*-value |
| **DII** |  |  |  |  |  |  |  |  |  |  |  |
| Continuous | 1.07(1.06, 1.09) | <0.001 |  | 1.10(1.08, 1.12) | <0.001 |  | 1.06(1.04, 1.07) | <0.001 |  | 1.05(1.03, 1.07) | <0.001 |
| Quartile |  |  |  |  |  |  |  |  |  |  |  |
| Q1 | 1 (Reference) |  |  | 1 (Reference) |  |  | 1 (Reference) |  |  | 1 (Reference) |  |
| Q2 | 0.94(0.85, 1.03) | 0.182 |  | 1.01(0.92, 1.11) | 0.789 |  | 1.00(0.91, 1.10) | 0.966 |  | 1.01(0.92, 1.11) | 0.782 |
| Q3 | 1.04(0.95, 1.14) | 0.413 |  | 1.13(1.03, 1.24) | 0.006 |  | 1.04(0.95, 1.15) | 0.355 |  | 1.04(0.95, 1.15) | 0.354 |
| Q4 | 1.36(1.25, 1.48) | <0.001 |  | 1.55(1.42, 1.69) | <0.001 |  | 1.29(1.18, 1.41) | <0.001 |  | 1.27(1.16, 1.39) | <0.001 |
| *P* for trend |  | <0.001 |  |  | <0.001 |  |  | <0.001 |  |  | <0.001 |
| **E-DII** |  |  |  |  |  |  |  |  |  |  |  |
| Continuous | 1.30(1.25, 1.35) | <0.001 |  | 1.37(1.31, 1.42) | <0.001 |  | 1.22(1.17, 1.27) | <0.001 |  | 1.20(1.16, 1.26) | <0.001 |
| Quartile |  |  |  |  |  |  |  |  |  |  |  |
| Q1 | 1 (Reference) |  |  | 1 (Reference) |  |  | 1 (Reference) |  |  | 1 (Reference) |  |
| Q2 | 0.98(0.89, 1.08) | 0.715 |  | 1.02(0.93, 1.13) | 0.655 |  | 1.00(0.91, 1.11) | 0.947 |  | 0.99(0.90, 1.10) | 0.901 |
| Q3 | 1.08(0.98, 1.18) | 0.132 |  | 1.16(1.05, 1.27) | 0.003 |  | 1.07(0.98, 1.18) | 0.143 |  | 1.07(0.97, 1.17) | 0.176 |
| Q4 | 1.59(1.45, 1.73) | <0.001 |  | 1.76(1.61, 1.92) | <0.001 |  | 1.43(1.31, 1.57) | <0.001 |  | 1.41(1.28, 1.54) | <0.001 |
| *P*for trend |  | <0.001 |  |  | <0.001 |  |  | <0.001 |  |  | <0.001 |

Continuous: Hazard ratio for per increase of 1 in DII or E-DII

Model 1 represents the single-factor Cox regression model (DII or E-DII)

Model 2 adjusted model 1 + age, sex, ethnicity, education, and TDI.

Model 3 adjusted model 2 + smoking status, alcohol intake frequency, physical activity and sleep duration.

Model 4 adjusted model 3 + BMI, hypertension, diabetes, hyperlipidemia and asthma.

Abbreviations: CI, confidence interval; DII, dietary inflammatory index; E-DII, energy-adjusted dietary inflammatory index; HR, hazard ratio; Q, quartile.

**Supplementary Table 3.** Association between DII, E-DII and risk of COPD using competitive risk model (N = 167,440).

| Index | Model 1 | |  | Model 2 | |  | Model 3 | |  | Model 4 | |
| --- | --- | --- | --- | --- | --- | --- | --- | --- | --- | --- | --- |
|  | HR (95% CI) | *P*-value |  | HR (95% CI) | *P*-value |  | HR (95% CI) | *P*-value |  | HR (95% CI) | *P*-value |
| **DII** |  |  |  |  |  |  |  |  |  |  |  |
| Continuous | 1.07(1.06, 1.09) | <0.001 |  | 1.10(1.08, 1.12) | <0.001 |  | 1.05(1.04, 1.07) | <0.001 |  | 1.05(1.03, 1.07) | <0.001 |
| Quartile |  |  |  |  |  |  |  |  |  |  |  |
| Q1 | 1 (Reference) |  |  | 1 (Reference) |  |  | 1 (Reference) |  |  | 1 (Reference) |  |
| Q2 | 0.94(0.86, 1.03) | 0.170 |  | 1.01(0.92, 1.11) | 0.790 |  | 1.00(0.91, 1.10) | 0.960 |  | 1.01(0.92, 1.11) | 0.770 |
| Q3 | 1.04(0.95, 1.13) | 0.450 |  | 1.13(1.03, 1.24) | 0.009 |  | 1.04(0.95, 1.14) | 0.430 |  | 1.03(0.94, 1.13) | 0.480 |
| Q4 | 1.36(1.25, 1.48) | <0.001 |  | 1.53(1.41, 1.67) | <0.001 |  | 1.27(1.17, 1.39) | <0.001 |  | 1.25(1.15, 1.37) | <0.001 |
| *P* for trend |  | <0.001 |  |  | <0.001 |  |  | <0.001 |  |  | <0.001 |
| **E-DII** |  |  |  |  |  |  |  |  |  |  |  |
| Continuous | 1.30(1.24, 1.35) | <0.001 |  | 1.36(1.30, 1.41) | <0.001 |  | 1.21(1.16, 1.26) | <0.001 |  | 1.19(1.14, 1.24) | <0.001 |
| Quartile |  |  |  |  |  |  |  |  |  |  |  |
| Q1 | 1 (Reference) |  |  | 1 (Reference) |  |  | 1 (Reference) |  |  | 1 (Reference) |  |
| Q2 | 1.01(0.92, 1.11) | 0.850 |  | 1.05(0.96, 1.16) | 0.300 |  | 1.03(0.94, 1.13) | 0.520 |  | 1.02(0.93, 1.12) | 0.670 |
| Q3 | 1.08(0.99, 1.19) | 0.091 |  | 1.16(1.06, 1.27) | 0.002 |  | 1.08(0.98, 1.19) | 0.110 |  | 1.07(0.98, 1.18) | 0.150 |
| Q4 | 1.61(1.48, 1.75) | <0.001 |  | 1.76(1.62, 1.92) | <0.001 |  | 1.43(1.31, 1.56) | <0.001 |  | 1.40(1.28, 1.53) | <0.001 |
| *P* for trend |  | <0.001 |  |  | <0.001 |  |  | <0.001 |  |  | <0.001 |

Continuous: Hazard ratio for per increase of 1 in DII or E-DII

Model 1 represents the single-factor Cox regression model (DII or E-DII)

Model 2 adjusted model 1 + age, sex, ethnicity, education, and TDI.

Model 3 adjusted model 2 + smoking status, alcohol intake frequency, physical activity and sleep duration.

Model 4 adjusted model 3 + BMI, hypertension, diabetes, hyperlipidemia and asthma.

Abbreviations: CI, confidence interval; DII, dietary inflammatory index; E-DII, energy-adjusted dietary inflammatory index; HR, hazard ratio; Q, quartile.

**Supplementary Table 4.** The association of DII, E-DII with COPD risk after excluding participants with asthma at baseline (N = 149,308).

| Index | Model 1 | |  | Model 2 | |  | Model 3 | |  | Model 4 | |
| --- | --- | --- | --- | --- | --- | --- | --- | --- | --- | --- | --- |
|  | HR (95% CI) | *P*-value |  | HR (95% CI) | *P*-value |  | HR (95% CI) | *P*-value |  | HR (95% CI) | *P*-value |
| **DII** |  |  |  |  |  |  |  |  |  |  |  |
| Continuous | 1.07(1.05, 1.09) | <0.001 |  | 1.10(1.08, 1.12) | <0.001 |  | 1.05(1.03, 1.07) | <0.001 |  | 1.05(1.03, 1.07) | <0.001 |
| Quartile |  |  |  |  |  |  |  |  |  |  |  |
| Q1 | 1 (Reference) |  |  | 1 (Reference) |  |  | 1 (Reference) |  |  | 1 (Reference) |  |
| Q2 | 0.93(0.83, 1.03) | 0.172 |  | 1.00(0.90, 1.12) | 0.939 |  | 0.99(0.89, 1.10) | 0.870 |  | 0.99(0.89, 1.11) | 0.893 |
| Q3 | 1.04(0.94, 1.16) | 0.417 |  | 1.15(1.04, 1.28) | 0.009 |  | 1.04(0.94, 1.15) | 0.474 |  | 1.03(0.93, 1.15) | 0.533 |
| Q4 | 1.36(1.23, 1.50) | <0.001 |  | 1.55(1.41, 1.72) | <0.001 |  | 1.26(1.14, 1.40) | <0.001 |  | 1.25(1.13, 1.39) | <0.001 |
| *P* for trend |  | <0.001 |  |  | <0.001 |  |  | <0.001 |  |  | <0.001 |
| **E-DII** |  |  |  |  |  |  |  |  |  |  |  |
| Continuous | 1.36(1.30, 1.43) | <0.001 |  | 1.37(1.31, 1.42) | <0.001 |  | 1.20(1.15, 1.26) | <0.001 |  | 1.20(1.16, 1.26) | <0.001 |
| Quartile |  |  |  |  |  |  |  |  |  |  |  |
| Q1 | 1 (Reference) |  |  | 1 (Reference) |  |  | 1 (Reference) |  |  | 1 (Reference) |  |
| Q2 | 1.02(0.91, 1.13) | 0.796 |  | 1.05(0.94, 1.17) | 0.375 |  | 1.03(0.92, 1.15) | 0.630 |  | 1.03(0.92, 1.15) | 0.610 |
| Q3 | 1.11(1.00, 1.24) | 0.052 |  | 1.19(1.07, 1.32) | 0.002 |  | 1.10(0.98, 1.22) | 0.102 |  | 1.10(0.99, 1.22) | 0.090 |
| Q4 | 1.60(1.45, 1.77) | <0.001 |  | 1.75(1.58, 1.94) | <0.001 |  | 1.39(1.26, 1.55) | <0.001 |  | 1.40(1.26, 1.56) | <0.001 |
| *P* for trend |  | <0.001 |  |  | <0.001 |  |  | <0.001 |  |  | <0.001 |

Continuous: Hazard ratio for per increase of 1 in DII or E-DII

Model 1 represents the single-factor Cox regression model (DII or E-DII)

Model 2 adjusted model 1 + age, sex, ethnicity, education, and TDI.

Model 3 adjusted model 2 + smoking status, alcohol intake frequency, physical activity and sleep duration.

Model 4 adjusted model 3 + BMI, hypertension, diabetes and hyperlipidemia.

Abbreviations: CI, confidence interval; DII, dietary inflammatory index; E-DII, energy-adjusted dietary inflammatory index; HR, hazard ratio; Q, quartile.

**Supplementary Table 5.** Association of DII and E-DII with the risk of COPD after multiple imputation of missing covariates (N = 208,255).

| Index | Model 1 | |  | Model 2 | |  | Model 3 | |  | Model 4 | |
| --- | --- | --- | --- | --- | --- | --- | --- | --- | --- | --- | --- |
|  | HR (95% CI) | *P*-value |  | HR (95% CI) | *P*-value |  | HR (95% CI) | *P*-value |  | HR (95% CI) | *P*-value |
| **DII** |  |  |  |  |  |  |  |  |  |  |  |
| Continuous | 1.08(1.07, 1.10) | <0.001 |  | 1.10(1.09, 1.12) | <0.001 |  | 1.06(1.04, 1.07) | <0.001 |  | 1.05(1.04, 1.07) | <0.001 |
| Quartile |  |  |  |  |  |  |  |  |  |  |  |
| Q1 | 1 (Reference) |  |  | 1 (Reference) |  |  | 1 (Reference) |  |  | 1 (Reference) |  |
| Q2 | 0.94(0.87, 1.02) | 0.144 |  | 1.01(0.93, 1.10) | 0.792 |  | 1.00(0.92, 1.08) | 0.947 |  | 1.01(0.93, 1.09) | 0.875 |
| Q3 | 1.06(0.98, 1.14) | 0.163 |  | 1.15(1.06, 1.24) | <0.001 |  | 1.05(0.97, 1.13) | 0.229 |  | 1.06(0.98, 1.14) | 0.177 |
| Q4 | 1.45(1.34, 1.55) | <0.001 |  | 1.60(1.49, 1.72) | <0.001 |  | 1.32(1.23, 1.42) | <0.001 |  | 1.31(1.22, 1.41) | <0.001 |
| *P* for trend |  | <0.001 |  |  | <0.001 |  |  | <0.001 |  |  | <0.001 |
| **E-DII** |  |  |  |  |  |  |  |  |  |  |  |
| Continuous | 1.31(1.27, 1.35) | <0.001 |  | 1.36(1.31, 1.40) | <0.001 |  | 1.21(1.17, 1.25) | <0.001 |  | 1.19(1.15, 1.23) | <0.001 |
| Quartile |  |  |  |  |  |  |  |  |  |  |  |
| Q1 | 1 (Reference) |  |  | 1 (Reference) |  |  | 1 (Reference) |  |  | 1 (Reference) |  |
| Q2 | 1.00(0.92, 1.08) | 0.926 |  | 1.03(0.95, 1.12) | 0.415 |  | 1.01(0.93, 1.09) | 0.843 |  | 1.00(0.92, 1.08) | 0.979 |
| Q3 | 1.12(1.03, 1.21) | 0.006 |  | 1.19(1.10, 1.29) | <0.001 |  | 1.10(1.01, 1.19) | 0.020 |  | 1.10(1.01, 1.19) | 0.026 |
| Q4 | 1.63(1.52, 1.77) | <0.001 |  | 1.77(1.64, 1.90) | <0.001 |  | 1.42(1.32, 1.53) | <0.001 |  | 1.40(1.29, 1.51) | <0.001 |
| *P*for trend |  | <0.001 |  |  | <0.001 |  |  | <0.001 |  |  | <0.001 |

Continuous: Hazard ratio for per increase of 1 in DII or E-DII

Model 1 represents the single-factor Cox regression model (DII or E-DII)

Model 2 adjusted model 1 + age, sex, ethnicity, education, and TDI.

Model 3 adjusted model 2 + smoking status, alcohol intake frequency, physical activity and sleep duration.

Model 4 adjusted model 3 + BMI, hypertension, diabetes, hyperlipidemia and asthma.

Abbreviations: CI, confidence interval; DII, dietary inflammatory index; E-DII, energy-adjusted dietary inflammatory index; HR, hazard ratio; Q, quartile.
